# Detection of variants in dystroglycanopathy-associated genes through the application of targeted whole-exome sequencing analysis to a large cohort of patients with unexplained limb-girdle muscle weakness

**DOI:** 10.1186/s13395-018-0170-1

**Published:** 2018-07-30

**Authors:** Katherine Johnson, Marta Bertoli, Lauren Phillips, Ana Töpf, Peter Van den Bergh, John Vissing, Nanna Witting, Shahriar Nafissi, Shirin Jamal-Omidi, Anna Łusakowska, Anna Kostera-Pruszczyk, Anna Potulska-Chromik, Nicolas Deconinck, Carina Wallgren-Pettersson, Sonja Strang-Karlsson, Jaume Colomer, Kristl G. Claeys, Willem De Ridder, Jonathan Baets, Maja von der Hagen, Roberto Fernández-Torrón, Miren Zulaica Ijurco, Juan Bautista Espinal Valencia, Andreas Hahn, Hacer Durmus, Tracey Willis, Liwen Xu, Elise Valkanas, Thomas E. Mullen, Monkol Lek, Daniel G. MacArthur, Volker Straub

**Affiliations:** 1 John Walton Muscular Dystrophy Research Centre, Institute of Genetic Medicine, Newcastle University, Newcastle upon Tyne, UK; 2 Newcastle Hospitals NHS Foundation Trust, Newcastle upon Tyne, UK; 3 Neuromuscular Reference Centre, University Hospital St-Luc, University of Louvain, Brussels, Belgium; 4 Copenhagen Neuromuscular Center, Rigshospitalet, University of Copenhagen, Copenhagen, Denmark; 5 Iranian Center for Neurological Research, Shariati Hospital, Tehran University of Medical Sciences, Tehran, Iran; 6 Department of Neurology, Medical University of Warsaw, Warsaw, Poland; 7 Ghent University Hospital, De Pintelaan 185, Ghent, Belgium; 8 Paediatric Neurology Department, Hôpital Universitaire des Enfants Reine Fabiola, ULB, Brussels, Belgium; 9 The Folkhaelsan Department of Medical Genetics, The Folkhaelsan Institute of Genetics, and the Department of Medical and Clinical Genetics, University of Helsinki, Topeliuksenkatu 20, Helsinki, Finland; 10 Children’s Hospital, Helsinki University Hospital and University of Helsinki, Helsinki, Finland; 11 Unitat de Patología Neuromuscular, Servei de Neurologia, Hospital Sant Joan de Déu, Barcelona, Spain; 12 Department of Neurology, University Hospitals Leuven, Leuven, Belgium; 13 Laboratory for Muscle Diseases and Neuropathies, Department of Neurosciences, University of Leuven (KU Leuven), Leuven, Belgium; 14 Department of Neurology and Institute of Neuropathology, RWTH Aachen University Hospital, Aachen, Germany; 15 Neurogenetics Group, VIB-UA, Center for Molecular Neurology, University of Antwerp, Antwerp, Belgium; 16 Laboratory of Neuromuscular Pathology, Institute Born-Bunge, University of Antwerp, Antwerp, Belgium; 17 Neuromuscular Reference Centre, Department of Neurology, Antwerp University Hospital, Antwerp, Belgium; 18 Abteilung Neuropädiatrie, Medizinische Fakultät Carl Gustav Carus, Technische Universität Dresden, Dresden, Germany; 19 Neuroscience Area, Biodonostia Health Research Institute, San Sebastián, Spain; 20 Center for Biomedical Research in the Neurodegenerative Diseases (CIBERNED) Network, Instituto Carlos III, Ministry of Economy and Competitiveness, Madrid, Spain; 21 Servicio de Neurología, Hospital de Mendaro, Osakidetza, Mendaro, Spain; 22 Department of Child Neurology, Justus-Liebig University, Gießen, Germany; 23 Department of Neurology, Istanbul Faculty of Medicine, Istanbul University, Istanbul, Turkey; 24 The Robert Jones and Agnes Hunt Orthopaedic Hospital, NHS Foundation Trust, Oswestry, UK; 25 Analytic and Translational Genetics Unit, Massachusetts General Hospital, Boston, MA USA; 26 Program in Medical and Population Genetics, Broad Institute of MIT and Harvard, Cambridge, MA USA

**Keywords:** Whole-exome sequencing, Dystroglycanopathies, Limb-girdle muscle weakness

## Abstract

**Background:**

Dystroglycanopathies are a clinically and genetically heterogeneous group of disorders that are typically characterised by limb-girdle muscle weakness. Mutations in 18 different genes have been associated with dystroglycanopathies, the encoded proteins of which typically modulate the binding of α-dystroglycan to extracellular matrix ligands by altering its glycosylation. This results in a disruption of the structural integrity of the myocyte, ultimately leading to muscle degeneration.

**Methods:**

Deep phenotypic information was gathered using the PhenoTips online software for 1001 patients with unexplained limb-girdle muscle weakness from 43 different centres across 21 European and Middle Eastern countries. Whole-exome sequencing with at least 250 ng DNA was completed using an Illumina exome capture and a 38 Mb baited target. Genes known to be associated with dystroglycanopathies were analysed for disease-causing variants.

**Results:**

Suspected pathogenic variants were detected in *DPM3*, *ISPD*, *POMT1* and *FKTN* in one patient each, in *POMK* in two patients, in *GMPPB* in three patients, in *FKRP* in eight patients and in *POMT2* in ten patients. This indicated a frequency of 2.7% for the disease group within the cohort of 1001 patients with unexplained limb-girdle muscle weakness. The phenotypes of the 27 patients were highly variable, yet with a fundamental presentation of proximal muscle weakness and elevated serum creatine kinase.

**Conclusions:**

Overall, we have identified 27 patients with suspected pathogenic variants in dystroglycanopathy-associated genes. We present evidence for the genetic and phenotypic diversity of the dystroglycanopathies as a disease group, while also highlighting the advantage of incorporating next-generation sequencing into the diagnostic pathway of rare diseases.

**Electronic supplementary material:**

The online version of this article (10.1186/s13395-018-0170-1) contains supplementary material, which is available to authorized users.

## Background

*DAG1* (dystroglycan 1) is a 5.8 kb gene transcript on chromosome 3p21 that is widely expressed in human tissue, including in the skeletal muscle [1]. The protein undergoes *N*- and *O*-linked glycosylation and is post-transcriptionally cleaved into two mature subunits: the ~ 156 kDa secreted cell surface α-dystroglycan and the 43 kDa transmembrane β-dystroglycan [2]. The dystroglycan complex, composed of one α- and one β-subunit, is a component of the larger dystrophin-glycoprotein complex that links the subsarcolemmal actin cytoskeleton to the extracellular matrix (ECM) [3]. ECM ligands such as laminin strictly rely on the correct presentation of glycan structures on the surface of α-dystroglycan for binding [4], and without this precise glycosylation, the function and integrity of the muscle cell are compromised.

Alpha-dystroglycan is glycosylated by a series of tightly regulated proteins as it is processed from the nucleus, through the endoplasmic reticulum and Golgi apparatus to reach the cell surface [5, 6]. Mutations in any of the genes encoding these proteins could result in diminished protein function, aberrant α-dystroglycan glycosylation and thus a reduced capacity to bind ECM ligands [7]. The disorders associated with abnormal α-dystroglycan glycosylation are collectively known as dystroglycanopathies. The clinical manifestations of dystroglycanopathies are extremely variable with a spectrum of severity within the disease group [8, 9]. Severe dystroglycanopathies are congenital and result in structural brain, eye and muscle abnormalities such as Walker-Warburg syndrome [10], muscle-eye-brain disease [11] and Fukuyama muscular dystrophy [12]. The spectrum then ranges through to limb-girdle muscular dystrophies (LGMD), which constitute less severe forms of the disease group with an adult onset and no brain or eye abnormalities [13, 14].

Homozygous and compound heterozygous mutations in 17 genes, plus *DAG1* itself, have been associated with dystroglycanopathies [15]. The encoded proteins include those with well-defined glycosyltransferase functions as well as those with less well-characterised functions (Table 1). In addition to researching those already known to be associated with dystroglycanopathies, another challenge is to identify additional proteins in the glycosylation pathway that underlie disease pathology. For example, it is estimated that 20–50% of suspected dystroglycanopathy patients do not harbour mutations in characterised dystroglycanopathy genes, suggesting the genetic cause of the disease is currently unknown for approximately half of patients. With such genetic and clinical heterogeneity among dystroglycanopathies, the causative genes can be difficult to determine from the phenotypes alone.

**Table 1:** Genes associated with dystroglycanopathies

Gene	Protein function	Associated dystroglycanopathy according to OMIM [49]	Reference
*B3GALNT2*	Glycosyltransferase	Congenital muscular dystrophy-dystroglycanopathy type A 11, with brain and eye anomalies	[50]
*B3GNT1*	Glycosyltransferase	Congenital muscular dystrophy-dystroglycanopathy type A 13, with brain and eye anomalies	[51]
*DAG1*	Connects actin cytoskeleton to extracellular matrix	Congenital muscular dystrophy-dystroglycanopathy type A 9, with brain and eye anomalies	[52]
		Limb-girdle muscular dystrophy-dystroglycanopathy type C 9	[53]
*DOLK*	Kinase	Congenital disorder of glycosylation type Im	[54]
*DPM1*	Transferase	Congenital disorder of glycosylation type Ie	[55]
*DPM2*	Transferase	Congenital disorder of glycosylation type Iu	[56]
*DPM3*	Transferase	Congenital disorder of glycosylation type Io	[44]
*FKRP*	Glycosyltransferase	Limb-girdle muscular dystrophy-dystroglycanopathy type C 5/LGMD2I	[25]
		Congenital muscular dystrophy-dystroglycanopathy type A 5, with or without mental retardation	[57]
		Congenital muscular dystrophy-dystroglycanopathy type B 5, with or without mental retardation	[25]
*FKTN*	Glycosyltransferase	Congenital muscular dystrophy-dystroglycanopathy type A 4, with brain and eye anomalies	[58]
		Congenital muscular dystrophy-dystroglycanopathy type B 4, without mental retardation	[28]
		Limb-girdle muscular dystrophy-dystroglycanopathy type C 4	[59]
*GMPPB*	Transferase	Congenital muscular dystrophy-dystroglycanopathy type A 14, with brain and eye anomalies	[30]
		Congenital muscular dystrophy-dystroglycanopathy type B 14, with mental retardation	
		Limb-girdle muscular dystrophy-dystroglycanopathy type C 14	
*ISPD*	Synthase	Congenital muscular dystrophy-dystroglycanopathy type A 7, with brain and eye anomalies	[47]
		Limb-girdle muscular dystrophy-dystroglycanopathy type C 7	[60]
*LARGE1*	Glycosyltransferase	Congenital muscular dystrophy-dystroglycanopathy type B 6, with mental retardation	[61]
*POMGNT1*	Glycosyltransferase	Congenital muscular dystrophy-dystroglycanopathy type A 3, with brain and eye anomalies	[62]
		Congenital muscular dystrophy-dystroglycanopathy type B 3, with mental retardation	[28]
		Limb-girdle muscular dystrophy-dystroglycanopathy type C 3	[63]
*POMGNT2*	Glycosyltransferase	Congenital muscular dystrophy-dystroglycanopathy type A 8, with brain and eye anomalies	[64]
*POMK*	Kinase	Congenital muscular dystrophy-dystroglycanopathy type A 12, with brain and eye anomalies	[65]
		Limb-girdle muscular dystrophy-dystroglycanopathy type C 12	[66]
*POMT1*	Glycosyltransferase	Congenital muscular dystrophy-dystroglycanopathy type A 1, with brain and eye anomalies	[67]
		Congenital muscular dystrophy-dystroglycanopathy type B 1, with mental retardation	[68]
		Limb-girdle muscular dystrophy-dystroglycanopathy type C 1	[69]
*POMT2*	Glycosyltransferase	Congenital muscular dystrophy-dystroglycanopathy type A 2, with brain and eye anomalies	[29]
		Congenital muscular dystrophy-dystroglycanopathy type B 2, with mental retardation	
		Limb-girdle muscular dystrophy-dystroglycanopathy type C 2	
*TMEM5*	Glycosyltransferase	Congenital muscular dystrophy-dystroglycanopathy type A 10, with brain and eye anomalies	[45]

Next-generation sequencing (NGS) is a well-developed methodology that can benefit the understanding, characterisation and diagnosis of such rare neuromuscular diseases. The entire exome of a patient can be sequenced, known as whole-exome sequencing (WES), at rapidly declining costs. Immediately performing WES could negate the need for undirected preliminary molecular investigations that can inflate the costs of a diagnostic evaluation [16]. MYO-SEQ is a research collaboration between academia, patient organisations and industry. This project applies WES to patients with unexplained limb-girdle muscle weakness and targets the analysis towards genes with a known association to neuromuscular diseases. Included in the candidate gene list were those involved in the glycosylation of α-dystroglycan. Here, we present the identification of 27 patients with putative pathogenic variants in dystroglycanopathy-associated genes from the targeted WES analysis of 1001 patients with unexplained limb-girdle muscle weakness. We highlight the diversity of the disease group and offer further characterisations of the diseases at the milder, limb-girdle weakness end of the spectrum.

## Methods

### Patients

Ethical approval (REC reference number 08/H0906/28) was granted by the Newcastle and North Tyneside Research Ethics Committee. One thousand and one patients were recruited throughout Europe and neighbouring countries, and informed written consent was given by all. Inclusion criteria stipulated that patients must present with limb-girdle muscle weakness and/or elevated serum creatine kinase (CK) activity.

### Whole-exome sequencing and data analysis

WES and data processing were performed by the MacArthur laboratory at the Broad Institute of MIT and Harvard (Broad Institute, Cambridge, MA, USA) as detailed previously [17]. The variant call set was uploaded onto the MacArthur laboratory’s *seqr* platform for genomic data analysis. The biological relevance of the detected variants was determined by considering the (i) ClinVar reports of pathogenicity [18], (ii) published literature, (iii) population frequency detailed by the gnomAD reference population database from the Broad Institute that contains exome data from over 120,000 individuals [19] and (iv) deleteriousness of the variant predicted by PolyPhen-2 [20], SIFT [21], MutationTaster2 [22] and FATHMM [23]. The detected variants were matched to the patient’s phenotype, and those that were most likely to be disease-causing were reported back to the referring clinician.

### Muscle histopathology

Muscle biopsies were obtained for all patients and analysed following standard histological techniques for light microscopy by local pathologists. Biopsy reports from pathology departments formed the basis to describe histological features in our patient cohort. Immunostaining was performed for 21 patients. The primary anti-α-dystroglycan antibodies that were used were VIA4-1 and IIH6C4 (Millipore), and NBP2-14868 (Novus Biologicals) for patients 6 and 7 only. The techniques used for patients 9, 10, 13 and 23 were unknown.

## Results

### Homozygous variants within dystroglycanopathy-associated genes

Dystroglycanopathies have so far only been reported with an autosomal recessive pattern of inheritance, and so only homozygous or compound heterozygous mutations in the associated genes could result in the disease phenotype, while heterozygous carriers are not affected by muscle disease. Of the 1001 patients (46% female, 54% male, mean age 39 years, median age 38 years, age range 2 to 88 years) whose exomes were analysed, nine patients had suspected pathogenic homozygous variants that could account for their clinical presentations (Table 2). Patient 1 had a rare homozygous missense variant in *DPM3* (p.Leu44Pro) that is absent in homozygosity in the control population and that affects a highly conserved amino acid [24]. At the age of 30 years, he presented with, and fully recovered from, an asymmetric brachial plexopathy (serum creatine kinase [CK] activity of 4310 IU/L [normal < 200 IU/L]). Twelve years later, he displayed an unsteady gait (serum CK levels of 2732 IU/L), and a proximal lower limb weakness mildly progressed over the course of the next 5 years. Patients 2–8 had homozygous missense variants in *FKRP* (p.Leu276Ile for all but patient 3 who instead had a p.Tyr182His change) [25], with a young age at disease onset, proximal muscle weakness and an elevated serum CK activity. Patient 7 first presented with muscle cramps on exercise. Patient 22 had a novel homozygous missense variant in *POMT2* (p.Gly238Val) [26], with an onset in childhood of proximal upper and lower limb weakness, central and cortical atrophy and spinal rigidity.

**Table 2:** Suspected pathogenic homozygous variants detected by the MYO-SEQ project in dystroglycanopathy-associated genes

Patient	Gene	Location			Predicted deleteriousness				ClinVar clinical significance	gnomAD allele frequency
		hg19 co-ordinates	Protein change	Sequence change	SIFT	PolyPhen-2	MutationTaster2	FATHMM		
1	*DPM3*	chr1:155112676	p.Leu44Pro	c.131T>C	Damaging	Probably damaging	Disease causing	Tolerated	No	0.000016^a^
2	*FKRP*	chr19:47259533	p.Leu276Ile	c.826C>A	Tolerated	Benign	Disease causing	Damaging	Pathogenic; likely pathogenic	0.001089^a^
3	*FKRP*	chr19:47259251	p.Tyr182His	c.544T>C	–	Probably damaging	Disease causing	Damaging	Uncertain	0.000018^a^
4	*FKRP*	chr19:47259533	p.Leu276Ile	c.826C>A	Tolerated	Benign	Disease causing	Damaging	Pathogenic; likely pathogenic	0.001089^a^
5	*FKRP*	chr19:47259533	p.Leu276Ile	c.826C>A	Tolerated	Benign	Disease causing	Damaging	Pathogenic; likely pathogenic	0.001089^a^
6	*FKRP*	chr19:47259533	p.Leu276Ile	c.826C>A	Tolerated	Benign	Disease causing	Damaging	Pathogenic; likely pathogenic	0.001089^a^
7	*FKRP*	chr19:47259533	p.Leu276Ile	c.826C>A	Tolerated	Benign	Disease causing	Damaging	Pathogenic; likely pathogenic	0.001089^a^
8	*FKRP*	chr19:47259533	p.Leu276Ile	c.826C>A	Tolerated	Benign	Disease causing	Damaging	Pathogenic; likely pathogenic	0.001089^a^
22	*POMT2*	chr14:77767536	p.Gly238Val	c.713G>T	Tolerated	Possibly damaging	Disease causing	Damaging	Uncertain	0.000000^a^

^a^Not reported in homozygosity in gnomAD

### Compound heterozygous variants within dystroglycanopathy-associated genes

**Fig. 1:**
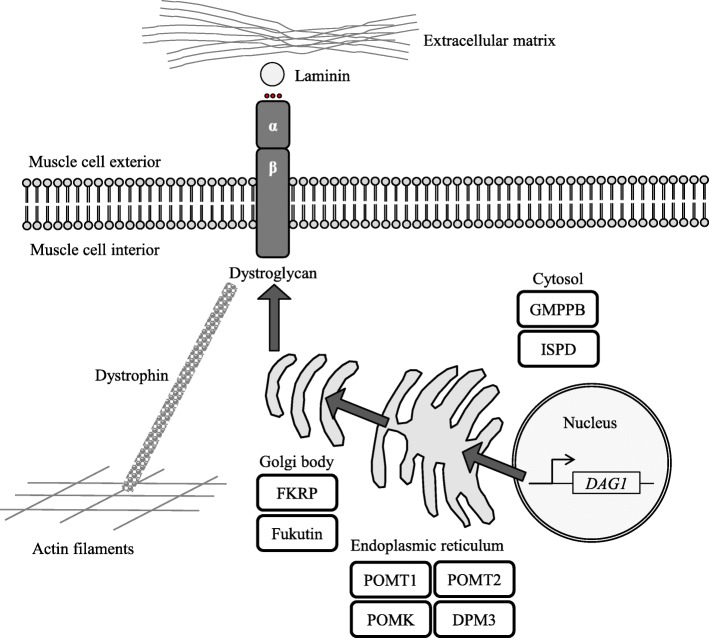
Localisation of the proteins involved in the glycosylation of α-dystroglycan. Only the encoded proteins of the genes identified as harbouring suspected pathogenic variants in the MYO-SEQ project are shown. *DAG1* is transcribed and translated into α-dystroglycan and β-dystroglycan subunits. As the proteins are processed through the endoplasmic reticulum and Golgi body to the muscle cell membrane (pathway indicated by grey arrows), GMPPB, POMT1, POMT2, POMK, ISPD, DPM3, FKRP and fukutin all contribute to the correct glycosylation of the α-subunit. The glycosylation of α-dystroglycan is required for interactions with extracellular matrix components; the dystroglycan complex as a whole thus acts as an anchor between the extracellular matrix and the intercellular actin cytoskeleton

An additional 18 patients had suspected pathogenic compound heterozygous variants in dystroglycanopathy-associated genes (Table 3). Patient 9 harboured variants in *FKRP* (p.Leu276Ile and p.Pro462Ser) [25, 27] and had a largely similar phenotype to the homozygous *FKRP* patients. Patient 10 carried variants in *FKTN* (p.Arg307Ter and p.Arg307Gln) [28, 29], with infantile onset, limb-girdle weakness and elevated serum CK activity. Patients 11 and 12 harboured variants in *GMPPB* (p.Asp27His and p.Arg287Trp) [30, 31]. Patient 13 similarly carried the p.Asp27His missense variant in *GMPPB* [30], but instead, this occurred in combination with a novel frameshift variant (p.Met64Ter). All three patients had an onset of symptoms at a young age and a slowly progressive disease course. Patient 14 harboured variants in *ISPD* (p.Cys56ValfsTer60 and p.Ser202Leu), presenting with a slowly progressive limb-girdle muscle weakness phenotype, with both an EMG and a muscle biopsy displaying myopathic changes and an elevated serum CK activity. Onset was in young adulthood, and there were no ocular or cognitive impairments. Patients 15 and 16 were siblings from non-consanguineous parents who presented during childhood with muscle cramps, hyperlordosis, calf pseudohypertrophy, scapular winging and elevated serum CK activity [32]. They carried rare variants in *POMK* (p.Pro322Leu and p.Arg46Ter). Patient 17 carried a rare missense variant in *POMT1* (p.Pro66Leu) in addition to a frameshift variant (p.Asp723GlyfsTer8) [33]. He had a non-progressive disease course from infancy with limb-girdle weakness; he did not have any contractures or spinal rigidity. Patients 19–21 and patients 23–27 all harboured previously unreported variants in *POMT2*, seven of which were absent in the control population. Patient 23 carried novel frameshift (p.Gly705GlufsTer31) and missense (p.Thr184Met) [34] variants in *POMT2*, while patient 24 harboured two missense variants in the gene (p.Tyr666Cys and p.Tyr136His) [35]. A summary of the glycosylation pathway and the localisation of the associated proteins is depicted in Fig. 1.

**Table 3:** Suspected pathogenic compound heterozygous variants detected by the MYO-SEQ project in dystroglycanopathy-associated genes

Patient	Gene	Location			Predicted deleteriousness				ClinVar clinical significance	gnomAD allele frequency
		hg19 co-ordinates	Protein change	Sequence change	SIFT	PolyPhen-2	MutationTaster2	FATHMM		
9	*FKRP*	chr19:47259533	p.Leu276Ile	c.826C>A	Tolerated	Benign	Disease causing	Damaging	Pathogenic; likely pathogenic	0.001089^a^
		chr19:47260091	p.Pro462Ser	c.1384C>T	Damaging	Probably damaging	Disease causing	Damaging	No data	0.000009^a^
10	*FKTN*	chr9:108380248	p.Arg307Ter	c.919C>T	No data	No data	Disease causing	No data	Pathogenic; likely pathogenic	0.000020^a^
		chr9:108380249	p.Arg307Gln	c.920G>A	No data	Probably damaging	Disease causing	Damaging	Pathogenic	0.000012^a^
11	*GMPPB*	chr3:49761081	p.Asp27His	c.79G>C	Damaging	Possibly damaging	Disease causing	Damaging	Pathogenic	0.000655
		chr3:49759490	p.Arg287Trp	c.859C>T	Damaging	Possibly damaging	Disease causing	Tolerated	Pathogenic	0.000094^a^
12	*GMPPB*	chr3:49761081	p.Asp27His	c.79G>C	Damaging	Possibly damaging	Disease causing	Damaging	Pathogenic	0.000655
		chr3:49759490	p.Arg287Trp	c.859C>T	Damaging	Possibly damaging	Disease causing	Tolerated	Pathogenic	0.000094^a^
13	*GMPPB*	chr3:49760844	p.Met64Ter	c.190delA	No data	No data	No data	No data	No data	0.000000^a^
		chr3:49761081	p.Asp27His	c.79G>C	Damaging	Possibly damaging	Disease causing	Damaging	Pathogenic	0.000655
14	*ISPD*	chr7:16460782	p.Cys56ValfsTer60	c.165dupG	No data	No data	No data	No data	No data	0.000000^a^
		chr7:16415796	p.Ser202Leu	c.605C>T	Damaging	Probably damaging	Disease causing	Damaging	No data	0.000033^a^
15	*POMK*	chr8:42977932	p.Pro322Leu	c.965C>T	Damaging	Probably damaging	Disease causing	Tolerated	No data	0.000004^a^
		chr8:42958827	p.Arg46Ter	c.136C>T	No data	No data	Disease causing	No data	No data	0.000180^a^
16	*POMK*	chr8:42977932	p.Pro322Leu	c.965C>T	Damaging	Probably damaging	Disease causing	Tolerated	No data	0.000004^a^
		chr8:42958827	p.Arg46Ter	c.136C>T	No data	No data	Disease causing	No data	No data	0.000180^a^
17	*POMT1*	chr9:134381575	p.Pro66Leu	c.197C>T	Damaging	Probably damaging	Disease causing	Damaging	No data	0.000053^a^
		chr9:134398412	p.Asp723GlyfsTer8	c.2167dupG	No data	No data	No data	No data	Pathogenic	0.000171^a^
18	*POMT2*	chr14:77753158	p.Arg421Trp	c.1261C>T	Damaging	Probably damaging	Disease causing	Damaging	Likely pathogenic	0.000024^a^
		chr14:77762593	p.Thr344Pro	c.1030A>C	Damaging	Probably damaging	Disease causing	Damaging	Uncertain	0.000000^a^
19	*POMT2*	chr14:77746421	p.Leu577ProfsTer8	c.1727dupG	No data	No data	No data	No data	No data	0.000032^a^
		chr14:77753158	p.Arg421Trp	c.1261C>T	Damaging	Probably damaging	Disease causing	Damaging	Likely pathogenic	0.000024^a^
20	*POMT2*	chr14:77746421	p.Leu577ProfsTer8	c.1727dupG	No data	No data	No data	No data	No data	0.000032^a^
		chr14:77753158	p.Arg421Trp	c.1261C>T	Damaging	Probably damaging	Disease causing	Damaging	Likely pathogenic	0.000024^a^
21	*POMT2*	chr14:77751373	p.Gln499Arg	c.1496A>G	Damaging	Probably damaging	Disease causing	Damaging	No data	0.000000^a^
		chr14:77755120	p.Arg413Pro	c.1238G>C	Damaging	Probably damaging	Disease causing	Damaging	Pathogenic; uncertain	0.000028^a^
23	*POMT2*	chr14:77744748	p.Gly705GlufsTer31	c.2114_2135delGAATCCTGAGCCTGCTCCTGGG	No data	No data	No data	No data	No data	0.000000^a^
		chr14:77769283	p.Thr184Met	c.551C>T	No data	Probably damaging	Disease causing	Damaging	Pathogenic	0.000007^a^
24	*POMT2*	chr14:77772712	p.Tyr136His	c.406T>C	Damaging	Probably damaging	Disease causing	Damaging	No data	0.000000^a^
		chr14:77745107	p.Tyr666Cys	c.1997A>G	Damaging	Probably damaging	Disease causing	Damaging	Pathogenic; likely pathogenic	0.000061^a^
25	*POMT2*	chr14:77767432	ESS	c.816+1G>A	No data	No data	Disease causing	No data	No data	0.000004^a^
		chr14:77778319	p.Phe102Leu	c.306C>A	Damaging	Probably damaging	Disease causing	Damaging	No data	0.000000^a^
26	*POMT2*	chr14:77750156	p.His546Pro	c.1637A>C	No data	No data	Disease causing	No data	No data	0.000007^a^
		chr14:77769277	p.Cys186Tyr	c.1654-5T>G	Tolerated	Possibly damaging	Disease causing	Damaging	No data	0.000000^a^
27	*POMT2*	chr14:77746811	ExtSS	c.1654-5T>G	No data	No data	No data	No data	No data	0.000000^a^
		chr14:77765843	p.Leu293His	c.878T>A	No data	Probably damaging	Disease causing	Damaging	No data	0.000000^a^

*ESS* essential splice site, *ExSS* extended splice site

^a^Not reported in homozygosity in gnomAD

### Patient phenotypes

Overall, the 27 patients (18 compound heterozygotes and nine homozygotes) had an average age of 32 years (age range 11 to 63 years, median age 28 years), with 15 females (56%) and 12 males (44%) in the cohort. All patients had limb-girdle muscle weakness and an elevated serum CK activity (Additional file 1). The age of disease onset ranged from foetal to middle age, and while the majority of patients had a progressive disease, three patients had non-progressive symptoms. Forty-four percent of the 27 patients were unable to walk independently at the time of enrolment onto the project. Eighteen patients had normal cognitive capabilities; nine had reported cognitive impairment. Patients 13, 15 and 24 had slight valve defects, left ventricular enlargement and dilated cardiomyopathy, respectively, while patient 23 had a reduced left ventricular fractional shortening (LVFS) of 25%. The cardiac work-ups for the remaining patients were unremarkable. Fourteen patients had reduced forced vital capacities (FVC) ranging between 34 and 83%. Muscle imaging scans were available for 21 patients, four of which were unremarkable. The imaging for the remaining patients showed fatty replacement primarily of the paraspinal and proximal muscles of the lower limbs. Electromyography (EMG) results displayed a predominantly myopathic pattern, while muscle biopsies showed either myopathic and/or dystrophic changes. The biopsy of patient 6 showed internal nuclei and that of patient 8 showed glycogen accumulation. Immunohistochemistry (IHC) studies detected an α-dystroglycan deficiency or reduced α-dystroglycan glycosylation in 14 patients.

## Discussion

Eighteen genes are currently known to be associated with dystroglycanopathies, with more causal genes yet to be identified [15]. The genes and their specific mutations do not always correlate with the phenotype or its severity, meaning dystroglycanopathies are an inherently complex group of neuromuscular diseases. Here, we used an unbiased sequencing approach to investigate the underlying genetic mechanisms of this disease group. This was in the form of targeted WES, an NGS technology that interrogates the 1% of the human genome that is coding. As 85% of known pathogenic variants reside in the coding regions alone [36], both the cost and the analysis workload associated with a whole-genome sequencing approach were avoided. The success of WES is widely recognised and has been reported in a range of diseases such as Parkinson disease [37], autism [38] and inflammatory bowel disease [39]. Indeed, we used targeted WES analysis in 1001 patients with unexplained limb-girdle muscle weakness and have found suspected pathogenic variants in 27 (2.7%) patients across nine of the 18 dystroglycanopathy-associated genes. The patients did not harbour suspected or potentially pathogenic variants in any other of the 429 known neuromuscular disease genes that were analysed as part of our study. The absence of a unified international dystroglycanopathy registry means that such contributions to rare disease research may not be fully exploited, which would otherwise be advantageous in furthering current knowledge of the disease.

Despite the central presentation of proximal muscle weakness, which was shared by all of our patients, we did not observe a common, core phenotype that could reliably distinguish the disease group from other LGMDs. Phenotypic variability was high even within the same disease group; for example, the reported age of disease onset for patients with *POMT2* variants ranged from foetal to middle age. POMT2 deficiency was initially associated with Walker-Warburg syndrome, a congenital disease with brain, eye and muscle affections [40], while mutations in the gene were only later described to be associated with LGMD2N [34]. This pleiotropy could explain such phenotypic differences, where perhaps the functional severity of the mutations correlates with the extent of hypoglycosylation and thus the severity of the resulting phenotype.

Indeed, a correlation between reduced α-dystroglycan staining and clinical course was observed for patients with mutations in *POMT1*, *POMT2* and *POMGNT1* [41]. In contrast, however, the study did not always detect such a pattern for patients with mutations in *FKTN* and *FKRP*. Within our own cohort, staining for α-dystroglycan was performed for most of our patients, yet an α-dystroglycan deficiency or reduced α-dystroglycan glycosylation was only detected in 14 of these. Seven patients had no indication of a dystroglycanopathy based on immunostaining. It must be noted that a standardised staining protocol was used for many, but not all, of these patients. Conversely, an additional 15 patients of the remaining 209 for whom immunostaining was reported within the overall MYO-SEQ cohort had an α-dystroglycan deficiency or reduced α-dystroglycan glycosylation, yet none of these harboured suspected pathogenic variants in any known dystroglycanopathy genes. In fact, nine of these patients had suspected pathogenic variants in other neuromuscular disease genes, leaving only six currently undiagnosed patients. Together, this suggests that (i) the detection of changes in α-dystroglycan and its glycosylation may not be entirely accurate and/or uniform across different clinical sites, (ii) the glycosylation of α-dystroglycan may not be the only affected dystroglycanopathy pathway, and (iii) other dystroglycanopathies are most likely yet to be characterised. Accordingly, the quantification of hypoglycosylation should not be the only gold standard for diagnosing the diseases. We propose that a complementary approach including NGS should be used to aid the correct diagnosis of these rare disease patients.

Our findings offer an expansion of the phenotypic spectrum of dystroglycanopathies and suggest that an unbiased NGS approach such as WES should be used for a reliable diagnosis. Dolichol-phosphate-mannose (DPM) synthase comprises the DPM1, DPM2 and DPM3 subunits and is required for the synthesis of a donor substrate (DPM) of glycosylation [42, 43]. A homozygous *DPM3* mutation (p.Leu85Ser) was first identified in an 11-year-old female with a mild LGMD, cardiomyopathy at 20 years of age and a stroke-like episode at 21 years of age [44]. Interestingly, our identification of a patient with a homozygous *DPM3* mutation (p.Leu44Pro) is the first description of such a patient without cardiomyopathy or central nervous system involvement [24]. Moreover, we identified two siblings with *POMK* mutations. Typically, patients with mutations in this gene have displayed a severe phenotype ranging from Walker-Warburg syndrome to LGMD with cognitive impairment. Unlike a previously described patient with *POMK* compound heterozygous variants, the siblings did not display a typical Walker-Warburg syndrome with cognitive impairments [45]. Finally, *FKTN* mutations are commonly associated with Fukuyama congenital muscular dystrophy in Japan, which is characterised by hypotonia, muscle weakness, and cerebral and cerebellar cortical dysplasia [12]. Fewer patients with LGMD caused by *FKTN* mutations have been reported, and these usually have a milder phenotype of proximal muscle weakness and no cognitive involvement [46]. Our Caucasian patient, as expected, did not carry the Japanese founder haplotype and displayed a slowly progressive limb-girdle and axial myopathy. This complements and enhances the current understanding of diseases associated with *FKTN* mutations. Compound heterozygous and homozygous mutations in *ISPD* have been associated with a broad clinical spectrum ranging from mild LGMD to a more severe Walker-Warburg syndrome [47], with the mutations predicted to be deleterious to protein function. However, patient 14 has only one frameshift variant that could result in a null allele and disrupt ISPD function (p.Cys56ValfsTer60); the second variant (p.Ser202Leu) is in an exon at the N terminal of the gene that is present in only one transcript. This may explain the observed milder phenotype of the patient.

We have observed that within our cohort, suspected pathogenic mutations in *POMT2* are the most common cause of dystroglycanopathies. This was similarly observed in a concentrated cohort of patients with α-dystroglycan hypoglycosylation [29]. Crucially, 15 of our 27 patients had suspected pathogenic variants in genes that were omitted from the aforementioned study (*DPM3*, *FKRP*, *GMPPB*, *ISPD* and *POMK*), indicating that an unbiased approach must be sought to fully characterise dystroglycanopathies. Indeed, even with a targeted NGS methodology, many of the patients in our MYO-SEQ cohort remain without a diagnosis. This suggests that patients may harbour pathogenic variants in genes with no currently known association to muscle disease, including uncharacterised dystroglycanopathies. It must be noted that rather than mutations in *POMT2*, those in *FKRP* are widely considered to cause the commonest dystroglycanopathy in Europe, LGMD2I [48]. While our data do not reflect this exactly, the 30% of patients with *FKRP* mutations strongly support the high prevalence of LGMD2I. A small *n* number, simple ascertainment bias and the ease at which *FKRP* can be sequenced in diagnostic work-ups—negating the need for inclusion in MYO-SEQ in these patients—could be responsible for these skewed data. Moreover, *FKRP* typically has a lower coverage with exome sequencing, meaning fewer LGMD2I patients might be detected relative to those identified through Sanger sequencing.

## Conclusions

Overall, dystroglycanopathies are a clinically and genetically heterogeneous group of disorders, and as such, accurate diagnoses are often difficult to achieve. We have collected a unique group of 27 dystroglycanopathy patients that offer an extended understanding of the phenotypes at the milder end of the disease spectrum. The disease group is highly diverse and complex, reinforced by the detection of variants in patients with an atypical dystroglycanopathy phenotype. We present evidence of the benefit of integrating WES into healthcare systems in an effort to diagnose rare diseases more readily and accurately. Further tests will now be required to determine the mechanisms by which these proteins act and to confirm the dysregulation of α-dystroglycan glycosylation.

## Additional file

Additional file 1: Clinical presentations and phenotypes of the 27 MYO-SEQ index cases with suspected pathogenic variants in genes associated with dystroglycanopathies. Muscle pathology findings are defined as a replacement of muscle with fat on T1-weighted axial images. Radiology reports from referring centres are not standardised or quantitative. FVC = forced vital capacity; LVEF = left ventricular ejection fraction; LVFS = left ventricular fractional shortening; RNS = repetitive nerve stimulation. No indications = immunostaining was performed but was not suggestive of an α-DG deficiency. (XLSX 14 kb)

## Abbreviations

CK: Creatine kinase
DAG1: Dystroglycan 1
DPM: Dolichol-phosphate-mannose
ECM: Extracellular matrix
EGA: European Genome–phenome Archive
EMG: Electromyography
FVC: Forced vital capacities
IHC: Immunohistochemistry
LGMD: Limb-girdle muscular dystrophies
LVEF: Left ventricular ejection fraction
LVFS: Left ventricular fractional shortening
NGS: Next-generation sequencing
RNS: Repetitive nerve stimulation
WES: Whole-exome sequencing
